# Nanotechnological approaches in the treatment of schistosomiasis: an overview

**DOI:** 10.3762/bjnano.15.2

**Published:** 2024-01-03

**Authors:** Lucas Carvalho, Michelle Sarcinelli, Beatriz Patrício

**Affiliations:** 1 Laboratory of Parasitic Diseases, FIOCRUZ, Avenida Brasil, 4365, Rio de Janeiro, Brazil; 2 Post-Graduate Program in Industrial Pharmaceutical Technology, Farmanguinhos, Oswaldo Cruz Foundation (FIOCRUZ), Rio de Janeiro, RJ, Brazil; 3 Pharmaceutical and Technological Innovation Laboratory - Department of Physiological Sciences, Biomedical Institute, R. Frei Caneca, 94, Rio de Janeiro, Brazil

**Keywords:** delivery system, nanoformulation, nanotechnology, neglected diseases, praziquantel, schistosoma

## Abstract

Schistosomiasis causes over 200,000 deaths annually. The current treatment option, praziquantel, presents limitations, including low bioavailability and resistance. In this context, nanoparticles have emerged as a promising option for improving schistosomiasis treatment. Several narrative reviews have been published on this topic. Unfortunately, the lack of clear methodologies presented in these reviews leads to the exclusion of many important studies without apparent justification. This integrative review aims to examine works published in this area with a precise and reproducible method. To achieve this, three databases (i.e., Pubmed, Web of Science, and Scopus) were searched from March 31, 2022, to March 31, 2023. The search results included only original research articles that used nanoparticles smaller than 1 µm in the treatment context. Additionally, a search was conducted in the references of the identified articles to retrieve works that could not be found solely using the original search formula. As a result, 65 articles that met the established criteria were identified. Inorganic and polymeric nanoparticles were the most prevalent nanosystems used. Gold was the primary material used to produce inorganic nanoparticles, while poly(lactic-*co*-glycolic acid) and chitosan were commonly used to produce polymeric nanoparticles. None of these identified works presented results in the clinical phase. Finally, based on our findings, the outlook appears favorable, as there is a significant diversity of new substances with schistosomicidal potential. However, financial efforts are required to advance these nanoformulations.

## Introduction

Schistosomiasis is a disease common in tropical countries caused by trematodes from the genus Schistosoma. More than 220 million people are affected by this disease, in addition to 800 million at risk of infection [1–2]. Every year, 200 thousand deaths are caused by schistosomiasis, making it the third most devastating tropical disease in the world after malaria and intestinal parasitosis [3]. After penetration of the skin by the larval form (cercariae), the schistosomes mature and migrate through the lung to the liver, gut, or bladder, depending on the species, where they elicit a marked immune response. The adult *Schistosoma mansoni* worms mate in the liver and lay eggs in the mesenteric venules of the intestine [4]. Nowadays, the only treatment available for this disease consists of praziquantel (PZQ) [5].

Praziquantel is a class II compound according to the biopharmaceutical classification system (BCS), so it has low solubility and high permeability in the gastrointestinal tract [6]. This drug is affected by the first-pass effect on the liver, which also impacts its bioavailability [6]. Unfortunately, this makes PZQ ineffective against young forms of *Schistosoma mansoni*, leading to concerns about the emergence of resistant strains. Indeed, reports of resistance have been documented worldwide, prompting research for alternative treatments or new approaches to improve the characteristics of PZQ [7]. Additionally, due to the first passage effect, high doses of PZQ are required, resulting in large tablet sizes, making its administration challenging for children, as no PZQ pediatric formulation is distributed by the World Health Organization (WHO). Consequently, people split adult PZQ tablets to treat children, but the bitter taste of PZQ makes it difficult for them to adhere to treatment [8]. Moreover, high dosages of PZQ have been associated with side effects such as abdominal pain, nausea, and allergy [9]. In this context, nanotechnological tools are being investigated as potential solutions to address all these issues related to PZQ and bring new treatment alternatives [10].

Nanotechnology involves the creation and use of materials and technologies at the nanoscale, while nanomedicine focuses on the application of nanotechnology to treat, monitor, and prevent diseases [11]. Nanomedicine uses nanocarriers to enhance drug delivery by ensuring that drugs are delivered in appropriate amounts to specific target areas and remains in the body for the necessary duration [12]. As a result, nanoparticles have been utilized mainly as drug delivery systems in various parasitic diseases, including schistosomiasis, to improve bioavailability, therapeutic efficacy, and decrease adverse effect profiles of the drugs used to treat such illnesses [13].

A few recent reviews provide a general overview of how nanotechnological tools are used in schistosomiasis treatment. However, most of these published works are narrative reviews limited to a specific drug or nanoparticle categories. For instance, some reviews only focus on PZQ [14], while others solely showcase nanosystems for drug delivery [15]. Nonetheless, recent literature reveals several works that employ various drugs and utilize nanoparticles not only as delivery systems but also with intrinsic action. Moreover, some of the previous works were not so clear about the methodology followed to include and exclude articles in their narrative review.

That said, the purpose of this work is to produce an integrative review of the theme with a well-defined research methodology, highlighting the main nanoparticles and drugs used in the literature to treat schistosomiasis.

## Results and Discussion

**Figure 1:**
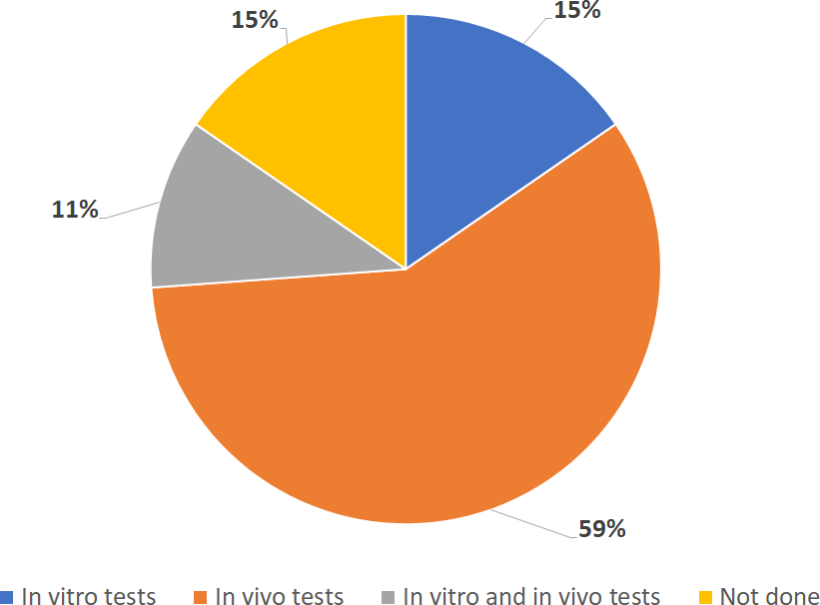
Percentage of articles that conducted effectiveness tests. The graph represents the number of articles out of the 65 found that conducted efficacy testing: solely in vitro, solely in vivo, both in vitro an in vivo, or none.

**Figure 2:**
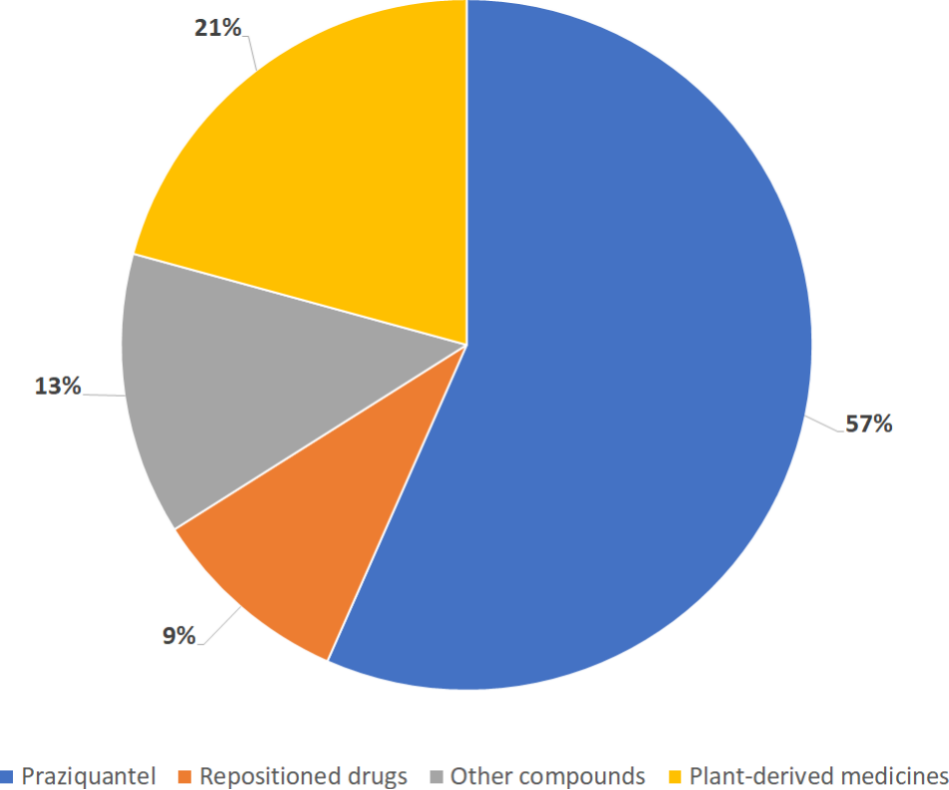
Percentage of compounds encapsulated among the selected articles. The graph considers a total of 53 articles that utilized nanosystems for drug delivery. It displays the percentage of these articles that used each drug category: praziquantel, plant-derived medicines, repositioned drugs, and other compounds.

**Figure 3:**
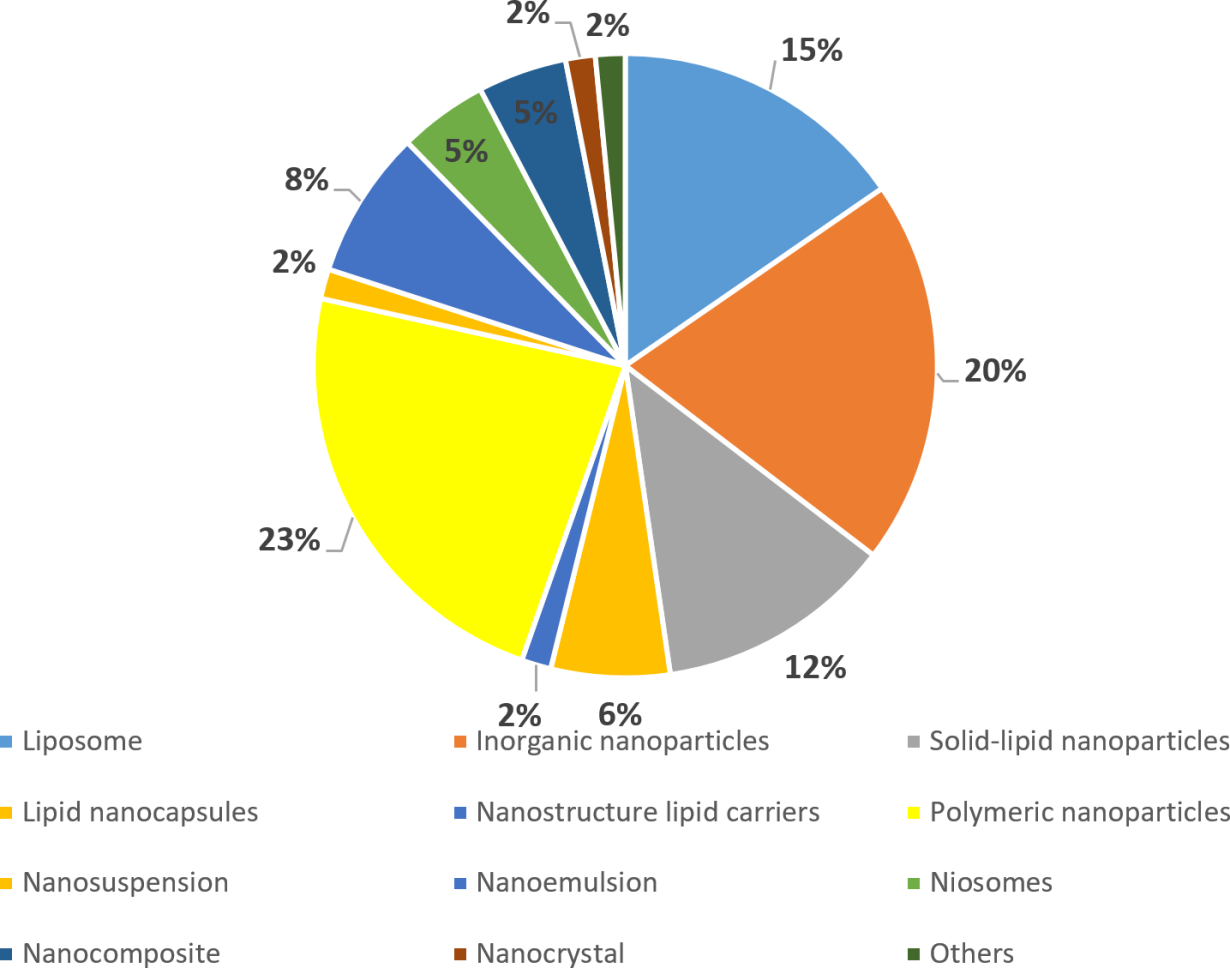
Percentage of types of nanosystems used in the selected articles. The graph considers a total of 65 articles found and displays the percentage of them that utilizes the differents nanosystems: liposomes, lipid nanocapsules, nanosuspensions, nanocomposites, inorganic nanoparticles, nanostructure lipid carriers, nanoemulsions, nanocrystals, solid lipid nanoparticles, polymeric nanoparticles, noisomes, and others.

**Figure 4:**
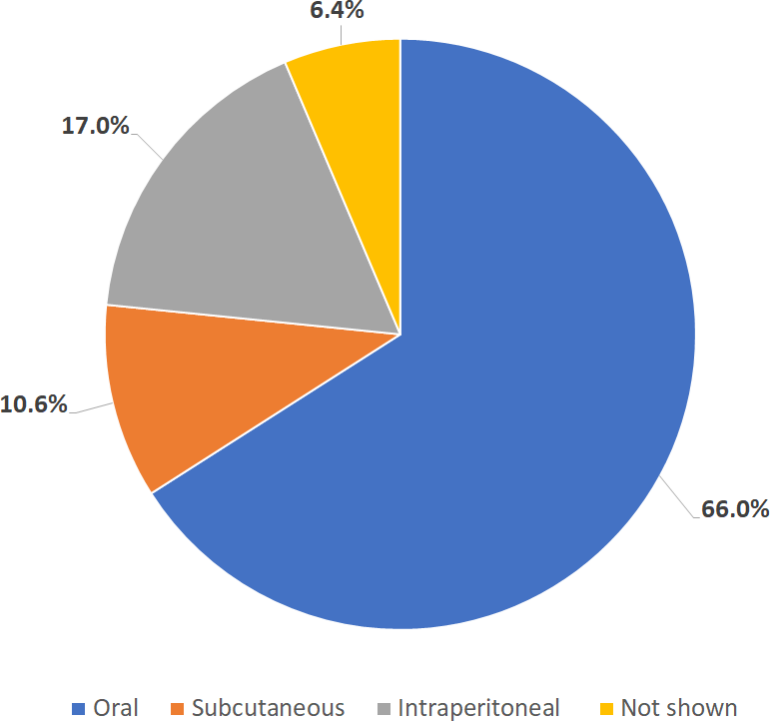
Percentage of administration routes used in the 65 selected articles. The routes were: oral, subcutaneous, or intramuscular. In some cases, this information was unclear in the articles.

We found 65 available articles that met the requirements, 75% (*n* = 49) were found in databases, while the remaining 25% (16 articles) were found through reference scanning. Table S1 (Supporting Information File 1) summarizes all the articles found regarding the use of nanosystems and encapsulated drugs. In Figure 1, it is possible to observe that only 59% of the publications show effectiveness data solely in vivo. Also, most articles use nanoparticles as drug delivery systems (82%), and most of them encapsulated PZQ (Figure 2). Polymeric (23%) and inorganic (20%) nanoparticles were used in the majority of the studies (Figure 3). Most of the papers (78%) have not done toxicity tests, and the main route of administration was the oral route (Figure 4).

It is important to bring attention to the fact that from the 65 papers found using our research strategy, 25% (16 articles) were found using reference scanning in the previously selected papers, which shows the importance of this step in bibliographic research. That explains why our strategy was able to reunite a great number of articles, unlike previous reviews. Below, we discuss the main findings of these studies.

### Nanosystems

Polymeric nanoparticles are nanoparticles composed of polymeric materials which may be natural or synthetic [16]. They are generally produced by two strategies: the dispersion of preformed polymers or the polymerization of monomers [17]. The first one is more commonly describe in the literature, and the techniques usually employed to produce them include nanoprecipitation, solvent evaporation, emulsification/solvent diffusion, and emulsification/reverse salting out [18–19]. The main advantages of using this type of nanoparticles as nanocarriers are their potential use for drug controlled release, the ability to protect drugs and other molecules with biological activity against the environment, improvement of their bioavailability and therapeutic index [17].

These nanocarriers are divided into two types: nanocapsules and nanospheres [18]. Nanocapsules consist of reservoir systems with an oil or water core and an external polymeric shell. They are, overall, used to increase drug solubility [20]. Usually, once in the body, the encapsulated drug diffuses through the polymeric wall in a zero-order kinetic, that means, it constantly releases the encapsulated drug [17]. In opposition, nanospheres are matrix systems formed by polymers without a central core. During the administration, the matrix erodes and the drug diffuses, resulting in a first-order kinetic drug release, that is, an exponential drug release [17,20].

Our research found that many articles utilized poly(lactic-*co*-glycolic acid) (PLGA) and chitosan nanoparticles, especially because they are biocompatible polymers and present great biodegradability. The polymer PLGA is approved for clinical use by Food and Drug Administration since 1989 [21] and, although no human data attests to chitosan safety, many animal tests prove its safety [22]. Eudragit L 100 is another polymer commonly used in literature because of its biocompatibility [8]. Overall, it is used when a delayed release is required. It is derived from polyacid, and because of that, it is resistant to low pH values [23]. So, when nanoparticles with this material are orally administered, they resist against gastric secretions and release the drug in the intestine. This protects many drugs and increases their bioavailability [24].

**Figure 5:**
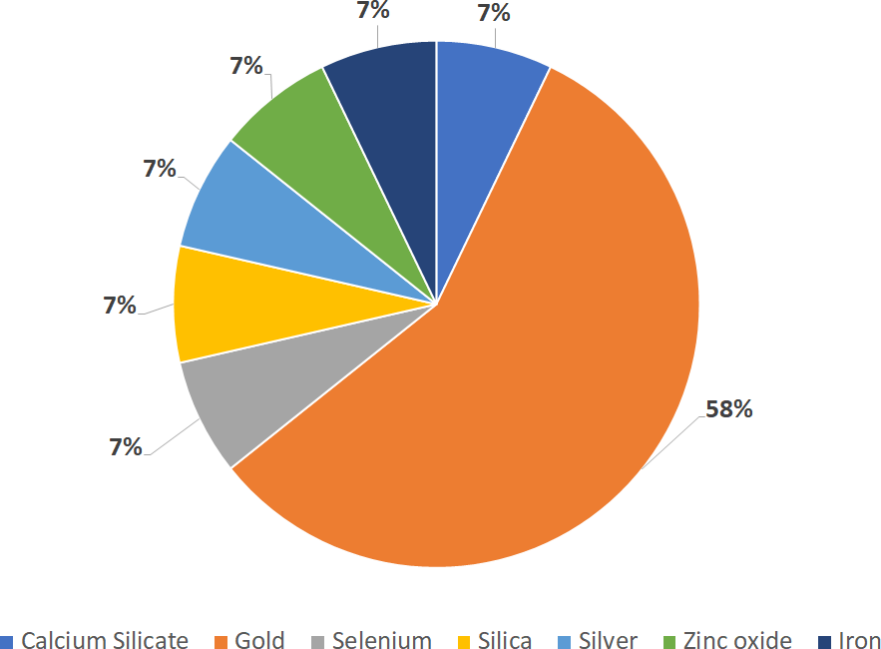
Percentage of material used to produce inorganic nanoparticles. The graph considers the 13 articles found that utilized inorganic nanoparticles and illustrates the type of material composing the nanoparticles used.

Inorganic nanoparticles (IN) are derived from metals or silica [25]. The most common metal used in the production of IN found in this work was gold, even though it was also possible to observe a great amount of works using silver and zinc (Figure 5). While silica-derived nanoparticles are used in the treatment against schistosomiasis due to their characteristics as drug carriers [10], metal nanoparticles usually present intrinsic action even when not loaded with drugs. Works with other parasites suggest that metallic nanoparticles may affect enzyme activity necessary to the physiology and production of the tegument [26]. Therefore, metallic nanoparticles also have a curative role against schistosomiasis. A possible explanation for this action was suggested by Dkhil et al. in their work with gold nanoparticles [27]. They suggested that their curative effects are due to antioxidant properties which confer the ability to scavenge free radicals [27]. After that, many authors related a reduction in oxidative stress markers in vivo after metalic nanoparticle administration and/or amelioration in histopathological characteristics after infection, which corroborates the first hypothesis [27–34].

Solid lipid nanoparticles (SLN) are solid lipid matrices at room and body temperature [35]. Their advantages are similar to classic nanocarriers, such as protection of labile drugs from biodegradation process, excellent excipient tolerability, and prolonged release. In addition, some disadvantages of the classic nanocarriers are not present in SLN, such as lack of biocompatibility, difficulty to produce on a large scale, and high raw material cost [36]. Many methods are used to prepare SLN, and they are divided into (1) high-energy methods, for dispersion of the lipid phase (such as high-pressure homogenization); (2) low-energy methods, which requires the precipitation of nanoparticles from homogeneous systems (such as microemulsions); and (3) methods based on organic solvents (emulsification–diffusion method) [35].

Liposomes are vesicles composed of a phospholipid and cholesterol with an aqueous core. It can have one or multiple layers. Due to that, their size can range from 30 nm to the micrometer range [37]. As drug vehicles, they exhibit unique properties, such as protection of encapsulated compounds from physiological degradation, extended drug half-life, controlled release of the drug molecule, and excellent biocompatibility and safety [38]. Liposomes can also be modified to selectively deliver a drug to a specific site. This is very valuable because it can reduce potential side effects and increase the maximum tolerated dose, which improves therapeutic benefits [39]. For example, Adekiya et al. [40] produced PZQ encapsulated in nanoliposomes whose surface was modified with an antibody against calpain, a protein found in the tegument of the parasite and is upregulated in the regions where host–parasite interaction occurs [41]. The modified nanoparticles orally administered two or four weeks postinfection altered the drug release pattern in vitro, were more efficient in reducing worm burden and the amount of eggs in the gut than PZQ alone, and altered the oogram pattern with the predominant presence of dead eggs. In addition, the nanoformulation showed no relevant toxicity in in vitro and in vivo models. Finally, the author discusses the possibility that the nanoformulation could be used to treat cases of schistosomiasis in the brain due to its smaller size [40]. Nevertheless, it is noteworthy that oral administration of biodegradable nanoparticles, such as conventional liposomes, exposes them to degradation by stomach acid, bile salts, and enzymes. Consequently, in in vivo models, intact liposomes may encounter challenges to reach the bloodstream owing to the adverse conditions of the stomach [42]. This elucidates why, in in vitro tests, the author exclusively assessed the release pattern of praziquantel by liposomes, omitting an examination of the impact of intact nanoparticles on the parasite, a facet explored by other researchers. Importantly, it is well known in the literature that modifications can be made to conventional liposomes to render these nanoparticles resistant to gastrointestinal barriers, representing another avenue of opportunity for the presented nanoformulation [42].

Niosomes are nanosystems similar to liposomes but formed using non-ionic surfactants like Span 60 [43]. They also could incorporate cholesterol in their structure beyond other lipids such as liposomes [44]. Therefore, they are able to be used as a carrier of amphiphilic or lipophilic drugs [45]. The main advantages of using this type of system are that they are osmotically active and stable and increase the stability of the entrapped drug. They could be used in oral, parenteral, and topical routes, and they are biodegradable, biocompatible, and non-immunogenic [45]. Moreover, they improve the therapeutic performance of the drug by protecting it from the biological environment and restricting effects to target cells, thereby reducing the clearance of the drug [45].

### Drugs for treatment of schistosomiasis

#### Praziquantel

It is not surprising that praziquantel is the most encapsulated drug. It remains the only effective frontline medicine to treat the disease, and is currently characterized by its exclusive and extensive use as an important antischistosomal drug [46]. However, PZQ also brings disadvantages, such as occurrence of resistant strains, low bioavailability [47], and organoleptic characteristics such as bitter taste [29,48]. In the literature, it is described that nanoformulations approaches can overcome these drawbacks.

Most publications used nanotechnology to alter pharmacokinetics parameters. The nanoformulations were evaluated through efficacy criteria (e.g., parasite burden, egg counts, and granuloma diameter) or using traditional pharmacokinetics parameters (e.g., absorption rate or bioavailability). For example, Labib El Gendy et al. [49] showed that PZQ encapsulated in liposomes (500 mg/kg) could be more efficient than free PZQ treatment. Similar results have been shown in other works that also used liposome with PZQ in different concentrations [50–53]. In addition, Xie et al. [54] studied the pharmacokinetics of solid lipid nanoparticles composed of castor oil encapsulating PZQ. They observed that the drug took more than one week in vitro to be released. A pharmacokinetic study in vivo also showed that the PZQ concentration in the plasma was sustained for longer times when the nanoformulation was studied in mice. Thus, the results show that solid lipid nanoparticles increase bioavailability in all administration routes tested (oral, subcutaneous, and intramuscular). However, results showed that subcutaneous delivery was superior to oral and intramuscular, promoting the longest therapeutic concentration in the circulation (264h) and the highest bioavailability.

There is just one formulation of PZQ developed for pediatric use which is commercially available: Epiquantel (40 mg/kg), a liquid formulation produced by Eipico, an Egyptian pharmacy industry. However, this medicine is not distributed by WHO and, thus, few works tried to use nanotechnology to change the organoleptic properties of PZQ [8,48]. da Fonseca et al. [8] used poly(methyl methacrylate) nanoparticles loaded with PZQ produced by in situ mini emulsion polymerizations to mask the drug taste and develop an oral formulation. Although the taste was masked, the authors reported a gritty tongue sensation caused by the high solid content of the formulation. In vitro results were satisfactory and showed that the nanoformulation was effective against parasites, but in vivo results were inadequate due to fluctuations in the administered dose. Despite that, the work showed that this nanoformulation could be used in the future [8]. In another work, Gonzalez et al. [48] increased the dissolution of PZQ by producing nanocrystals through high-pressure homogenization, followed by drying through spray-drying. After that, they resuspended the powder in Oral plus^®^ and Oral Sweet^®^, which are suspension vehicles known for their sweet taste and suitability for pediatric formulations [48].

Finally, few works tried to combine PZQ nanoformulations with other drugs/treatments [52,55]. Frezza et al. [52] tested PZQ-liposomes (oral route, 100 mg/kg) with hyperbaric oxygen and observed that it reduced the number of worms in mice. The combination also reduced the oviposition, changed the oogram pattern, and caused alteration in parasite tegument [52]. Eissa et al. [55] proved that a nanoformulation combining PZQ (250 mg/kg) and miltefosine (20 mg/kg) was efficient against all stages of the parasites, including juvenile forms. It was also noted alterations in parasite tegument and a reduction in granulomatous reactions in murine liver.

Although finding new forms to improve PZQ characteristics is essential, it would not solve the problem once only this drug is available to treat the disease and resistant strains are described. Therefore, finding new approaches with new drugs is crucial to raise treatment possibilities. Despite the fact that PZQ is the most encapsulated drug and most of the reviews about schistosomiasis only focus on it, we would like to bring to this review other drugs that are being studied.

#### Plant-derived drugs

After PZQ, most of the works in the literature involved plant-derived compounds. Guimarães et al. [56] tested the efficiency of epiisopiloturine in vitro and the best way to extract this molecule from leaves. Epiisopiloturine is an imidazole alkaloid found in jaborandi leaves (*Pilocarpus microphyllus*), which has known activity against adult, young, and egg forms of *Schistosoma mansoni* [57]. Since this is an apolar molecule with poor solubility, the author proposed a nanosystem using liposomes to make this molecule more useful in schistosomiasis therapy. The results showed that epiisopiloturine (300 µg/mL) has an effect in vitro, but it is not superior to PZQ. However, other nanotechnological approaches can potentialize the effect of the drug. Therefore, further studies should be made. Furthermore, the results showed that epiisopiloturine was not toxic to mice peritoneal cells, which is an encouraging prognosis for the development of future products [56].

Curcumin is a naturally yellow pigment obtained from the rhizomes of *Curcuma longa*. In the literature, many articles explore anti-inflammatory, antioxidant, antiviral, anti-infectious, and antitumoral properties of curcumin [58]. Mokbel et al. [59] showed that curcumin associated with a half-dose of PZQ and gold nanoparticles reduced the worm load in infected mice more than PZQ alone. This information is crucial since most side effects presented by patients who use PZQ could be avoided if there was a way to reduce the drug dose. Despite that, the combination could not reduce egg count more than that with PZQ alone. Nonetheless, the author affirms in the presentation of their results that the combination is more effective than the use of PZQ alone in this aspect [59].

Luz et al. [58] showed that curcumin encapsulated by polymeric nanoparticles could kill 100% of adult worms in vitro at a concentration of100 µM. Lower concentrations reduced motility and caused tegumental alterations and couple separation [58]. However, curcumin has low bioavailability and poor water solubility. Thus, Aly et al. [60] tried to increase its solubility and permeability through the cellular membrane by making a nanoemulsion of *Curcuma longa* extract (i.e., the curcumin plant source). The nanoemulsion showed an effect against adults of *S. mansoni* in vitro (especially males). This is an interesting finding because data from the literature reports that females are usually more susceptible to drug action than males. However, in this work, the death of females was only possible in a high concentration of the nanoformulation (100 µg/mL). Every dosage tested was also effective against young forms (esquistosomules) [60].

El-Menyawy et al. [61] used thymoquinone, a bioactive compound isolated from *Nigella sativa*, encapsulated in chitosan nanoparticles. The nanoformulation reduces the worm load in mice by 60% (predominantly female) and the number of couples found in vivo. Although the results showed a clear difference between control groups and the groups treated with nanoparticles, the author considered the results not good enough since other works showed a more prominent reduction [62]. Regarding egg counts in the liver and intestine, the nanoformulation was more efficient than blank particles which shows the relevance of nanoparticle for drug delivery. The histopathological exam also showed that nanoparticles could reduce the number and size of granulomas and diminish changes caused by infection. Although these results are very promising and interesting, the author does not mention the way in which the formulation was administered, which prevents a more critical analysis by the reader [61].

Elawamy et al. [63] also used *N. sativa* in their work, but instead of using one specific compound, they used the whole extract from this plant and encapsulated it in chitosan nanoparticles. The results showed that it is possible to diminish the worm load and change the oogram pattern in mice using the oral nanoformulation alone or with PZQ. Furthermore, the nanoparticle alone had a more significant effect than that for when the extract was administered with PZQ regarding granuloma formation, reducing the number and diameter of granulomas. Thus, *N. sativa* extract associated with chitosan nanoparticles may be a pharmacological strategy to replace PZQ or to help lower its dosage. However, the author admits in this work that no data proves the biological safety of using chitosan in a nanoformulation [63].

While on the subject, other extracts of vegetal sources were also studied to treat schistosomiasis by using a nanotechnological approach. A method using ultracentrifugation and ultrasonic dispersion produced ginger (*Zingiber officinale*) extract-derived nanoparticles which an average size of 238.3 nm [64]. The author justified his choice to use this kind of nanoparticles, claiming that they are less expensive than conventional ones, and in the literature, they were already used to treat inflammatory diseases [65–66]. Data proved that these nanoparticles orally administered in mice reduced the worm load, but not more than PZQ or mefloquine. However, when ginger-derived nanoparticles were combined with a half-dose of mefloquine, the reduction in worm load was 100% even in a short time of infection (6 weeks). This combination also causes a reduction of 100% in hepatic and intestinal egg counts in the same period, in addition to showing a hepatoprotective effect conserving the typical tissue structure. Regarding granuloma formation, the combination was also efficient, although another combination using ginger-derived nanoparticles and PZQ caused a more significant effect than the total dose of PZQ. In addition to this, ginger-derived nanoparticles alone or combined with other drugs were able to cause alterations in parasite tegument [64].

Another work used carvacrol, a monoterpene present in essential oils derived from plants such as *Origanum vulgare*. Besides being commonly used as a flavoring agent in food and cosmetics, it shows antimicrobial activity. Xavier et al. [67] reported that nanoemulsions with carvacrol orally administered were able to reduce worm burden and eggs in feces more than PZQ in the prepatent period (21 days post-infection). This impressive result shows that this nanoformulation is more efficient in juvenile forms. The author also suggests that the mechanism by which the nanoemulsion could reduce the worm burden is its antimicrobial activity, connecting changes in microbiota with the response to parasites. However, the mechanism of action of carvacrol remains unknown [67].

#### Repositioned drugs

Works utilizing compounds repositioned from other diseases have also been found in our search. Miltefosine, for example, is a drug created to treat cutaneous metastasis from mammary carcinomas [68]. After that, it was also approved to treat leishmaniasis [69–70], and in 2011, Eissa et al. [71] verified that the drug has activity against different forms of *S. mansoni* in vivo. After that, the same group, in 2015, developed lipid nanocapsules positively charged (cationic) and tested them with and without oleic acid as a membrane permeabilizer in the composition. Both nanoformulations were able to reduce the whole treatment of schistosomiasis in mice to one single oral dose (20 mg/kg) [72]. In 2016, it was shown that despite both nanoformulations being effective, the formulation without oleic acid was more effective when administered on the first day of infection. On the other hand, oleic acid nanocapsules were more effective when administered 21 days after infection [73]. Late in 2020, while cationic lipid nanocapsules were hemolytic [72], the same group tested lipid nanocapsules with oleic acid on the membrane and miltefosine (20 mg/kg) alone or combined with PZQ. They reported that nanosystems containing miltefosine with or without PZQ were potent (when orally administered in mice) against all forms of *S. mansoni*, including juvenile forms. These nanosystems caused alterations in parasite tegument and reduced granulomatous reaction in the liver [55].

When administered by oral route in mice, celecoxib, a traditional non-steroidal inhibitor of cyclo-oxygenase used as an anti-inflammatory, analgesic, and antipyretic drug, was also effective against juvenile forms of *S. mansoni* when associated with solid lipid nanoparticles causing damage to parasite tegument [74].

Spironolactone is a diuretic drug mainly used to treat hypertension. Abd El Hady showed in vitro that polymeric nanoparticles were able to confer a sustained biphasic release pattern in comparison with that of spironolactone alone. Moreover, they proved in mice that orally administered nanoformulation was efficient against *S. mansoni* infection and induced significant reduction in spleen, liver indices, and total worm count, and it induced decline in the hepatic and small intestinal egg load. Finally, it also caused extensive damage to adult worms on tegument and suckers, leading to the death of the parasites in less time compared to that for the drug alone, and improve liver pathology [75].

#### Other compounds

Some of the selected works used new synthetic compounds in their formulation for schistosomiasis treatment. For example, 2-(butylamino)-1-phenyl-1-ethanethiosulfuric acid (BphEA) is a compound with poor solubility in water, which has demonstrated potential to be used in schistosomiasis treatment. Araújo et al. [76] developed a cationic nanoemulsion to increase solubility. This nanoemulsion increases efficiency in vitro, causing the death of female worms within three hours, alterations in tegument within 48 hours, and reduced male worm motility. A hypothesis suggested by the author is that the charge of nanoemulsion interacts with a negatively charged group in the tegument of parasites, facilitating drug delivery [76].

Articles utilizing synthetic drugs that were once used to treat schistosomiasis but, for safety reasons, were discontinued have also been found. Tartar emetic, for example, was part of the first class of compounds used to treat schistosomiasis [77]. However, due to their low therapeutic index and the rise of less toxic new drugs, it was discontinued. de Melo et al. [78] proved that pegylated liposomes could reduce toxicity and mortality of tartar emetic in mice even in high concentrations (27 mg Sb/kg). Although the mortality was reduced, drug efficiency remains unaltered, especially when the nanoformulation was intraperitoneally administered [78]. However, it is known that oral route adhesion is better than the others tested in this work (intraperitoneal and subcutaneous). Therefore, drug dosage forms with these characteristics may present compliance issues and problems with commercialization. Thus, an interesting pathway could be testing the same nanoformulations but using the oral route. After antimonials such as tartar emetic, oxamniquine was released in the market, and with PZQ they remain as the drugs that can be used to treat schistosomiasis. However, signs of rising resistance to the drug slowed down the demand [78–82]. In 1997, Frézard and Melo [83] showed that liposomes with oxamniquine (10 mg/kg) subcutaneously applied efficiently reduce the worm load three to 14 days after infection (with a maximum reduction of 60%) in mice. These reports indicate that nanotechnological approaches may be a hope not only for PZQ or new compounds but also for bringing back improved versions of old medicines.

Amer et al. [43] used ubiquinol, a natural inhibitor of neutral magnesium-dependent sphingomyelinase, a key enzyme in sphingomyelin breakdown. This enzyme is essential because sphingomyelin is crucial in forming the outer leaflet of the tegumental lipid bilayer membrane in *Schistosoma mansoni* [43].

Araújo et al. [84] verified the activity of the sulfated polysaccharide α-ᴅ-glucan, a polysaccharide naturally found in extracts of lichen from *Ramalina celastri*. This work shows that liposomes with this carbohydrate could eliminate adult worms from infected mice 56 days post-infection when it was administered by the intraperitoneal route. The results also show that the nanoformulation reduced the number of eggs in feces of infected mice and hepatic granuloma in the liver. However, no difference between the nanoformulation and the controls was observed (sulfated polysaccharide α-ᴅ-glucan administered alone and empty liposomes). Furthermore, mice treated with sulfated polysaccharide α-ᴅ-glucan presented granulomas with histochemical markers, which could mean that this molecule stimulates the immunological system causing changes in membrane carbohydrates. Moreover, it raises the hypothesis that this change in the membrane molecule pattern is related to the reduction in granulomas. Finally, the author suggests that sulfated polysaccharide α-ᴅ-glucan could be used with other drugs with significantly higher effects against schistosomiasis, such as PZQ and oxamniquine, to stimulate the immunological system [83].

Oleic acid, a common unsaturated free fatty acid in the outer layer of human skin, is commonly used as a permeation promoter, inducing the disruption of the lipid structure of the membrane. de Oliveira et al. [85] showed in vitro that oleic acid encapsulated in polymeric nanoparticles could potentially be used in schistosomiasis treatment. Cytotoxicity assays confirm the compatibility of this fatty acid with biosystems, and in vitro results showed that nanoparticles reduced the time of action of free oleic acid in four to six hours. Oleic acid nanoparticles (50 µg/mL) caused 100% of mortality of adult worms in 24 hours, while neither empty nanoparticles nor raw oleic acid were able to yield the same mortality rate at the same time in vitro. Doses lower than 50 µg/mL were able to cause worm separation and reduce motility. Doses higher than 25 µg/mL reduced oviposition when incubated for 24 hours. The results also show that even sublethal doses can cause alterations in parasite tegument [85].

Bee venom comprises various pharmacologically active components, including melittin (constituting more than 50% of total proteins) and a mixture of enzymes, cell-lytic peptides, proteases, and bioactive amines [86]. This mixture has antioxidant, anticoagulant, anti-bacterial, immunostimulatory, and hepatotoxic protection properties [87–89]. Because of that, it has been used in traditional medicine to treat inflammation and pain [90]. Mohamed et al. [91] reported that bee venom administered in infected mice reduces worm burden, ova count/liver, and granuloma diameter [91]. However, high concentrations of bee venom increase hepatic granuloma diameter. Thus, Badr et al. [92] tried to develop a nanoformulation approach to minimize the side effects of bee venom treatment. Polymeric nanoparticles created in their work allowed a sustained release and caused extreme changes in parasite tegument. In vivo, nanoparticles could reduce worm load and granuloma diameter and induce new biliary ducts. Nanoformulation was more effective in adult females than in juvenile forms and adult males [92].

Following the aforementioned studies, the majority of them (92%) utilized nanoformulations administered via the oral route. This outcome is unsurprising, as despite potential drawbacks such as first-pass metabolism, reduced bioavailability, and drug degradation throughout the digestive tract, the oral route is widely accepted and minimally invasive [93]. Consequently, releasing a new alternative to PZQ via a different route may not be the most advisable option, as it may not be well-received by patients, leading to potential commercialization challenges associated with a less familiar or less convenient delivery method.

### Effectiveness tests

Effectiveness tests are important to demonstrate how powerful a drug is against the parasite. In previous sections it was detailed how certain studies demonstrated the effect of tested formulations. Overall, the parameters used to measure the in vitro efficacy of the treatment are reduction in mortality and mobility, couple separation, and tegument alterations. In vivo, the main criteria used is reducing worm burden, quantity and diameter of granuloma, eggs in feces, and oxidative stress markers (e.g., glutathione, nitrite/nitrate, and malondialdehyde).

Generally, articles that do not show effectiveness data use known drugs which have its effectivity attested, and aim to increase the dissolution of the drug in vitro [48,94]. Yang et al. [94] verified that PZQ nanocrystals had a more significant dissolution rate than that of the conventional drug due to the particle size and, consequently, it also showed a bioavailability improvement. That is because bioavailability of orally administered drugs depends on their ability to be absorbed in the gastrointestinal tract. For class II drugs (e.g., PZQ) the absorption process is limited by drug dissolution rate in gastrointestinal media. Therefore, enhancement of the dissolution rate of these drugs will present improved bioavailability [95].

Other works do not show effectiveness tests because they are focused on evaluating pharmacokinetics. Cong et al. [96] showed that PZQ nanoemulsion has sustained drug release for a long time, both in vitro and in vivo. Mishra et al. [97] demonstrated similar conclusions using PZQ associated with solid lipid nanoparticles. Malhado et al. [98] concluded that PZQ associated with PMMA nanoparticles could not improve the pharmacokinetic curve. In fact, the absorption of the encapsulated drug was three times lower than that for conventional PZQ.

Other works do not address effectiveness tests because they evaluate the impact of nanosystems in physiological/pathological changes caused by *S. mansoni*. Dkhil et al. [32] showed that metallic nanoparticles could decrease all intestinal changes caused by the parasite. The nanoparticles avoided weight gain in infected mice, increased glutathione levels, and reduced the levels of oxidative stress markers. This work showed that selenium nanoparticles were even more effective than PZQ, reducing inflammation signs in jejunal tissue and tissue injury levels similarly to PZQ. El-Shorbagy et al. [34] showed that the treatment with gold nanoparticles decreased the granuloma index, but with less effectiveness in comparison to PZQ at concentrations of 2.5, and 1.25 µM/mL. Overall, the nanoparticles exhibited antioxidant effects in vitro.

### Toxicity tests

Toxicity testing is essential to guarantee the safety of the treatment. Most articles have dealt with cytotoxicity testing in vitro or acute toxicity testing in vivo. Others deviated from traditional methods and used genotoxicity testing and mitochondrial metabolism evaluation to assess this. However, toxicity data were not reported in most of the articles. Although no explanation has been given in the articles regarding the absence of safety tests, there are possible reasons to explain why some tests are missing. Many articles use compounds that already have their safety stablished (e.g., PZQ or repurposed drugs) which have been approved before and their side effects are known. This was also the case in the Amer et al. [99] article in which ubiquinol, a natural compound approved as a dietary supplement, was used. Therefore, the safety tests were deemed unnecessary. Many papers that did not provide toxicity data concerning nanosystems referred to previous articles in which safety testing was performed. However, it is important to highlight that even nanosystems that were tested before must be tested again if the study uses a different experimentation design (different drug concentrations, different methods to produce nanoparticles, or a different therapeutical scheme). Numerous articles in our research have substantiated this information. For instance, the study conducted by Amara et al. [100] in 2018 demonstrated that diverse compositions of lipid nanocapsules resulted in varying IC_50_ values. Additionally, this research revealed that the IC_50_ value of encapsulated PZQ was considerably higher than that of PZQ administered alone, underscoring the significance of conducting toxicity testing even for well-known drugs. That means that part of the articles selected in this review still must prove the safety of their nanoformulations. This is the only way for the product to advance to the next stages, such as clinical phase.

In fact, none of the papers in this work was in clinical trials, reflecting the small number of nanosystems that enter the clinical phase. This probably happens not only because many of these works do not present safety data, but also because of the high costs of clinical trials [101]. As a neglected disease, schistosomiasis does not have the investment necessary by the private sector. Nevertheless, schistosomiasis remains a disease with a big economic impact, especially in underdeveloped and developing nations. For example, in 2015, its impact costs US$ 41,7 million to Brazil [102].

Moreover, it is imperative to address the additional complexities associated with nanoparticle formulations. While it is evident that manufacturing nanoparticles incurs high costs, it is essential to highlight other intricacies related to these formulations. Despite none of the authors explicitly mentioning stability challenges as a concern in the nanoparticle manufacturing process, especially in tropical regions characterized by elevated temperatures and humidity, it is a critical aspect to consider. Such environmental conditions pose formidable obstacles to the effective deployment of these formulations [103]. Furthermore, upscaling presents a significant issue. As demonstrated in previous discussions, many of the articles employed production techniques that are challenging to scale up, with batch-to-batch variations further complicating the manufacturing process [104]. As a result, achieving a consistent and reproducible manufacturing process becomes a daunting task in the realm of nanoparticle formulations.

Thus, regardless of the reasons for the challenges in bringing nanoformulations to the market, the responsibility falls on the government to make concerted efforts and provide the necessary support to overcome economic and other barriers. This support is crucial for aiding research institutions in introducing new products to the market, which can effectively mitigate the impact of the disease in those countries.

## Conclusion

In this review, we selected 65 papers using three databases: Pubmed, Scopus, and Web of Science; and the reference within the selected papers. This is a great number since none of the recent reviews have brought this amount of articles on this topic [13,15,105] together. This is due to the methodology used in this paper, which included a reference scanning stage, responsible for 25% of the articles found. Moreover, our strategy allowed us to include articles not included in any of the previous reviews, proving that our method is more inclusive.

Inorganic and polymeric nanoparticles are among the most widely utilized nanotechnological systems. Most research articles utilized gold nanoparticle as inorganic nanoparticles, while PLGA and chitosan are commonly utilized to produce polymeric nanoparticles due to its biocompatibility reported in various animal studies. However, there is currently a lack of data to support the safety of chitosan formulations for human use.

Most of the articles reported superior results to PZQ in preclinical tests; however, no article was found in clinical phase. One of the reasons for that is the low financial support to treat schistosomiasis since it is a neglected disease. Nonetheless, there is big diversity of solutions with great potential to be superior to PZQ using nanotechnological resources. However, governmental investment is necessary for these nanomedicines to achieve full potential.

## Experimental

**Figure 6:**
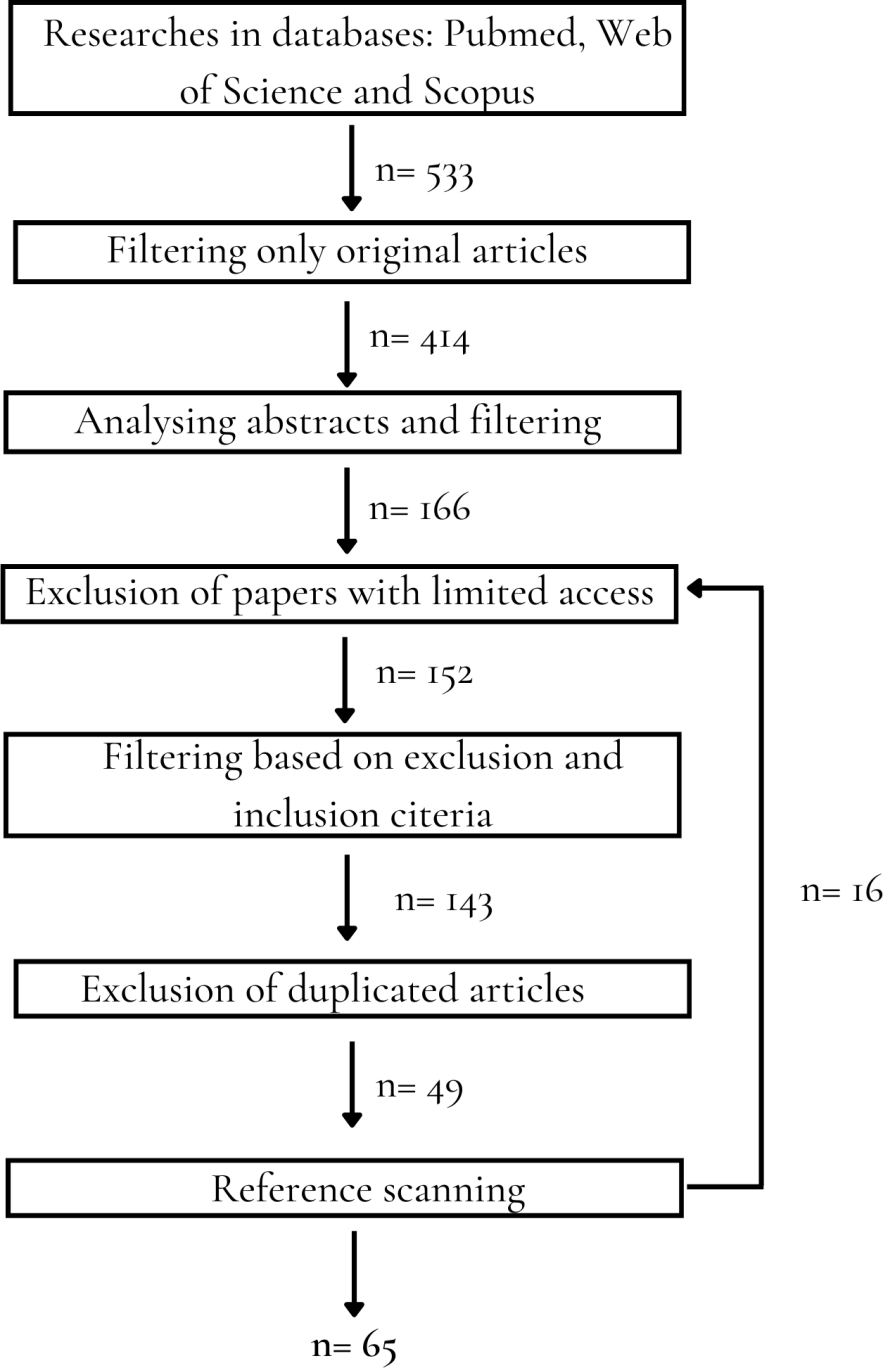
Methodology flow diagram. The search was conducted in three databases, and, following the selection steps shown, a total of 49 articles were found. Reference scanning was performed on these selected articles and 16 new articles were identified. Therefore, a total of 65 articles were included in this review.

Searches were done in Pubmed, Scopus, and Web of Science databases. These searches were conducted from March 31st, 2022, to March 31st, 2023, using the following search keywords: (nano* OR encapsul*) AND (treatment OR therap* OR activity OR chemotherapy) AND schistosomiasis. After obtaining the list of papers, a filter by type of article was applied, selecting only original research and excluding reviews. After that, the titles and abstracts were read, and articles unrelated to the theme were excluded. Afterward, it was checked if there was access to the remaining work. For those that could not be accessed, attempts were made to contact the authors and ask for a copy. The available articles were read entirely, and those unrelated to the theme were excluded. For exclusion, the criteria used were: (1) particle size over 999 nm; (2) articles that approach only prophylactic nanoformulations. After that, a search in the references of the selected papers was done to guarantee the maximal articles related to the theme in this review (Figure 6).

## Supporting Information

As supporting information we provide Table S1 cited in the results. This table shows the articles found using our methodology.

File 1 Nanosystems with their encapsulated drugs found in open access articles.
