# Supplementary material for: Nanotechnological approaches in the treatment of schistosomiasis: an overview
Source: Beilstein J Nanotechnol. 2024 Jan 3;15:13–25. doi: 10.3762/bjnano.15.2 (PMC10777326; doi:10.3762/bjnano.15.2)
Supplement: File 1 — Nanosystems with their encapsulated drugs found in open access articles. [file Beilstein_J_Nanotechnol-15-13-s001.pdf]

BEILSTEIN JOURNAL OF NANOTECHNOLOGY

Supporting Information

for

Nanotechnological approaches in the treatment of schistosomiasis: an overview

Lucas Carvalho, Michelle Sarcinelli and Beatriz Patrício

Beilstein J. Nanotechnol. **2024,** *15,* 13–25. doi:10.3762/bjnano.15.2

Nanosystems with their encapsulated drugs found in open access articles

License and Terms: This is a supporting information file under the terms of the Creative Commons Attribution License (https://creativecommons.org/licenses/by/4.0). Please note that the reuse, redistribution and reproduction in particular requires that the author(s) and source are credited and that individual graphics may be subject to special legal provisions.
The license is subject to the *Beilstein Journal of Nanotechnology* terms and conditions: (https://www.beilstein-journals.org/bjnano/terms)

Table S1: Nanosystems with their encapsulated drugs found in open access articles.

Nanosystem	Encapsulated drug	References
**Liposome**	Praziquantel	Mourão et al. 2005 [1]; Frezza et al. 2007 [2]; Frezza et al. 2013 [3]; Frezza et al. 2015 [4]; Labib El Gendy et al. 2019 [5]; Adekiya et al 2022a [6];
	Epiisopiloturine	Guimarães et al. 2014 [7];
	Sulfated Alpha-D-glucan	Araújo et al. 2011 [8]
	Tartar emetic	De Melo et al. 2003 [9]
	Oxamniquine	Frézard et al 1997 [10]
**Polymeric nanoparticles**	Praziquantel	Malhado et al. 2016 [11]; Paulino et al. 2022 [12]; Fonseca et al. 2013 [13]; Sadek et al. 2018 [14]; Mainardes et al. 2006 [15]; AboSheishaa et al 2019 [16].
	Thymoquinone	El-Menyawy et al. 2021 [17]; El-Menyawy et al 2022 [18].
	Oleic acid	Oliveira et al. 2021 [19]
	Bee venom	Badr et al. 2022 [20]
	Curcumin	Luz et al. 2012 [21]
	N. sativa extract	Elawamy et al 2019 [22];
	Licarin-A	Lima et al. 2022 [23];
	Spironolactone	Abd El Hady et al 2023 [24];
	Lignan-DNHK	Lima et al. 2017 [25]
**Solid-lipid nanoparticles**	Praziquantel	Radwan et al. 2019 [26]; de Souza et al. 2014 [27] ; Xie et al. 2010 [28]; Yang et al. 2009 [29]; AL-Noshokaty et al. 2020 [30]; Adekiya et al 2022b [31]; Mishra et al 2014 [32]
	Celecoxibe	Ibrahim et al. 2022 [33];
	Praziquantel	Eissa et al. 2020 [34]; Amara et al. 2018 [35];

**Lipid nanocapsules**	Milfetosine	Eissa et al 2020 [34]; Eissa et al. 2015 [36]; El-Molesmany et al. 2016 [37];
**Nanostructured lipid carriers**	Praziquantel	Kolenyak-Santos et al. 2015 [38]
**Niosomes**	Praziquantel	Zoghroban et al. 2019 [39]; Amer et al. 2022b [40]
	Ubiquinol	Amer et al. 2022a [41]
**Nanosuspension**	Praziquantel	Gonzalez et al. 2019 [42]
**Nanoemulsion**	Praziquantel	Cong et al. 2017 [43]
	Epiisopiloturine	Lima et al. 2018 [44]
	BphEA	Araújo et al. 2007 [45]
	Curcuma extract	Aly et al. 2017 [46]
	Carvacrol	Xavier et al. 2022 [47]
**Nanocomposite**	Praziquantel	El-Feky et al. 2015 [48]
	None	Bayaumy et al. 2016 [49]; Darwish et al. 2018 [50]
**Nanocrystal**	Praziquantel	Yang et al. 2019 [51]
**Inorganic nanoparticles**	Praziquantel	Tawfeek et al. 2019 [52]; Mokbel et al. 2020 [53]
	Curcumin	Mokbel et al. 2020 [53]
	None	Abou El-Nour et al. 2021 [54]; Dkhil et al. 2019 [55]; Dkhil et al 2015b [56]; Dkhil et al. 2017 [57]; Dkhil et al. 2016a [58]; Dkhil et al. 2016b [59]; Moustafa et al. 2018 [60]; Bauomy et al. 2020 [61]; El-Shorbagy et al. 2019 [62]; Khalil et al. 2018 [63]; Dkhil et al. 2015a [64]
**Others**	Ginger extract	Abd El Wahab et al. 2021 [65]

References

[1] Mourão, S. C., Costa, P. I., Salgado, H. R., Gremião, M. P. D. International journal of pharmaceutics **2005**, 295, 157-162. doi:10.1016/j.ijpharm.2005.02.009

[2] Frezza, T. F., Madi, R. R., Banin, T. M., Pinto, M. C., Souza, A. L. R., Gremião, M. P. D., Allegretti, S. M. Revista de Ciências Farmacêuticas Básica e Aplicada **2007**, 209-214.

[3] Frezza, T. F., Gremião, M. P. D., Zanotti-Magalhães, E. M., Magalhães, L. A., de Souza, A. L. R., Allegretti, S. M. Acta tropica **2013**, 128, 70-75. doi:10.1016/j.actatropica.2013.06.011

[4] Frezza, T. F., de Souza, A. L. R., Prado, C. C. R., de Oliveira, C. N. F., Gremião, M. P. D., Giorgio, S., Dolder, M. A. H., Joazeiro, P. P., Allegretti, S. M. Acta tropica **2015**, 150, 182-189. doi:10.1016/j.actatropica.2015.07.022

[5] Labib El Gendy, A. E. M., Mohammed, F. A., Abdel-Rahman, S. A., Shalaby, T. I. A., Fathy, G. M., Mohammad, S. M., El-Shafey, M., Mohammed, N. A. Journal of Parasitic Diseases **2019**, 43, 416-425. doi:10.1007/s12639-019-01106-6

[6] Arunachalam, A., Jeganath, S., Yamini, K., Tharangini, K. *International journal of novel trends in pharmaceutical sciences* **2012**, 2, 25-31.

[7] Guimarães, M. A., Campelo, Y. D., Veras, L., Colhone, M. C., Lima, D. F., Ciancaglini, P., Kuckelhaus, S. S., Lima, F. C. A., de Moraes, J., de Leite, J. R. S. A. *Journal of nanoscience and nanotechnology* **2014**, 14, 4519-4528. doi:10.1166/jnn.2014.8248

[8] Araújo, R. V. S., Melo-Júnior, M. R., Beltrão, E. I. C., Mello, L. A., Iacomini, M., Carneiro-Leão, A. M. A., Carvalho Jr, L. B., Santos Magalhães, N. S. *Brazilian Journal of Medical and Biological Research* **2011**, 44, 311-318. doi:10.1590/s0100-879x2011007500014

[9] de Melo, A. L., Silva-Barcellos, N. M., Demicheli, C., Frézard, F. *International journal of pharmaceutics* **2003**, 255, 227-230. doi:10.1016/s0378-5173(03)00125-x

[10] Frézard, F., Melo, A. L. D. *Revista do Instituto de Medicina Tropical de São Paulo* **1997**, 39, 97-100. doi:10.1590/S0036-46651997000200006

[11] Malhado, M., Pinto, D. P., Silva, A. C., Silveira, G. P., Pereira, H. M., Santos Jr, J. G., Guilarducci-Ferraz, C. V. V., Viçosa, A. L., Nele, M., Fonseca, L. B., Pinto, J. C. C. S., Calil-Elias, S. *Journal of pharmaceutical and biomedical analysis* **2016**, 117, 405-412. doi:10.1016/j.jpba.2015.09.023

[12] Paulino, É. T., Ribeiro de Lima, M., Viçosa, A. L., Silva, C. H. D., Salomon, C. J., Real, D. A., Leonardi, D., Silva, C. C. M., Moraes Neto, A. H. A. D. Frontiers in Public Health **2022**, 10, 848633. doi:10.3389/fpubh.2022.848633

[13] Fonseca, L. B., Viçosa, A. L., Mattos, A. C. A., Coelho, P. M. Z., Araújo, N., Zamith, H. D. S., Volpato, N. M., Nele, M., Pinto, J. C. C. S. *Vigil Sanitária Debate*, **2013**; 1, 82-88. doi:10.3395/vd.v1i4.111en

[14] Sadek, G. S., Harba, N., ELREFAI, S. A., SHARAF EL-DEEN, S. A., SALEH, M. M. *Journal of the Egyptian Society of Parasitology* **2018**, 48, 629-638.

[15] Mainardes, R. M., Chaud, M. V., Gremião, M. P. D., Evangelista, R. C. Journal of nanoscience and nanotechnology **2006**, 6, 3057-3061. doi:10.1166/jnn.2006.487

[16] AboSheishaa, G. A. Egyptian Veterinary Medical Society of Parasitology Journal (EVMSPJ) **2019**, 15, 149-162. doi:10.21608/evmspj.2019.145815

[17] El-Menyawy, H. M., Metwally, K. M., Aly, I. R., Abo Elqasem, A. A., Youssef, A. A. *The Egyptian Journal of Hospital Medicine* **2021**, 84, 1818-1826. doi:10.21608/EJHM.2021.177604

[18] El-Menyawy Et Al, H. M. Egyptian Journal of Aquatic Biology and Fisheries **2022**, 26, 1375-1392. doi:10.21608/EJABF.2022.268786

[19] de Oliveira, R. N., Campos, P. M., Pinto, R. M. C., Mioduski, J., Santos, R. D., Justus, B., de Paula, J. F. P., Klein, T., Boscardin, P. M. D., Corrêa, S. A. P., Allegretti, S. M., Ferrari, P. C. *Journal of Drug Delivery Science and Technology* **2021**, 63, 102429. doi:10.1016/j.jddst.2021.102429

[20] Badr, A. M., Saleh, A. H., Mahmoud, S. S., Mousa, M. R., Mahana, N. A., Dena, A. S. A. *Journal of Drug Delivery Science and Technology* **2022**, 71, 103344. doi:10.1016/j.jddst.2022.103344

[21] Luz, P. P., Magalhães, L. G., Pereira, A. C., Cunha, W. R., Rodrigues, V., Andrade e Silva, M. L. *Parasitology research* **2012**, 110, 593-598. doi:10.1007/s00436-011-2527-9

[22] Elawamy, W. E., Mohram, A. F., Naguib, M. M., Ali, H. S., Kishik, S. M., Hendawi, F. F. *Journal of Medicinal Plants Research* **2019**, 13, 443-451. doi:10.5897/JMPR2019.6842

[23] Lima, T. C., Magalhães, L. G., Paula, L. A. D. L., Cunha, W. R., Januário, A. H., Pauletti, P. M., Bastos, J. K., Mnuquian, H. A., Forim, M. R., Morais-Urano, R. P., Laurentiz, R. S., Tondato, W. N., Molina, E. F., Santos, M. F. C., e Silva, M. L. Experimental Parasitology **2022**, 241, 108357. doi:10.1016/j.exppara.2022.108357

[24] Abd El Hady, W. E., El-Emam, G. A., Saleh, N. E., Hamouda, M. M., Motawea, A. *International journal of nanomedicine* **2023**, 18, 987–1005. doi:10.2147/IJN.S389449

[25] Lima, T. C., Lucarini, R., Luz, P. P., de Faria, E. H., Marçal, L., Magalhães, L. G., Badoco, F. R., Esperandim, V. R., Molina, E. F., Laurentz, R. S., Lima, R. G., Cunha, W. R., Bastos, J. K., Silva, M. L. A. Pharmaceutical biology **2017**, 55, 2270-2276. doi:10.1080/13880209.2017.1405996

[26] Radwan, A., El-Lakkany, N. M., William, S., El-Feky, G. S., Al-Shorbagy, M. Y., Saleh, S., Botros, S. Parasites & vectors **2019**, 12, 1-12. doi:10.1186/s13071-019-3563-z

[27] de Souza, A. L. R., Andreani, T., de Oliveira, R. N., Kiill, C. P., dos Santos, F. K., Allegretti, S. M., Chaud, M. V., Souto, E. B., Silva, A. M., Gremião, M. P. D. International journal of pharmaceutics **2014**, 463, 31-37. doi:10.1016/j.ijpharm.2013.12.022

[28] Xie, S., Pan, B., Wang, M., Zhu, L., Wang, F., Dong, Z., Wang, X., Zhou, W. = *Nanomedicine* **2010**, 5, 693-701. doi:10.2217/nnm.10.42

[29] Yang, L., Geng, Y., Li, H., Zhang, Y., You, J., Chang, Y. Die Pharmazie-An International Journal of Pharmaceutical Sciences **2009**, 64, 86-89.

[30] AL-Noshokaty, T. M., Aly, I., Abo-Elmatty, D. M., Mesbah, N. M., Shehata, A. S., Etewa, S. Res J Parasitol **2020**; 15: 38-46.

[31] Adekiya, T. A., Kumar, P., Kondiah, P. P., Ubanako, P., Choonara, Y. E. International Journal of Molecular Sciences **2022b**, 23, 9485. doi:10.3390/ijms23169485

[32] Mishra, A., Vuddanda, P. R., Singh, S. *Journal of nanotechnology*, **2014**. doi:10.1155/2014/351693

[33] Ibrahim, E. I., Abou-El-Naga, I. F., El-Temsahy, M. M., Elsawy, E. S. A., Makled, S., Mogahed, N. M. F. H. *Acta Tropica* **2022**, 229, 106342. doi:10.1016/j.actatropica.2022.106342

[34] Eissa, M. M., El-Azzouni, M. Z., El-Khordagui, L. K., Abdel Bary, A., El-Moslemany, R. M., Abdel Salam, S. A. *Parasites & Vectors* **2020**, 13, 1-12. doi:10.1186/s13071-020-04346-1

[35] Amara, R. O., Ramadan, A. A., El-Moslemany, R. M., Eissa, M. M., El-Azzouni, M. Z., El-Khordagui, L. K. *International journal of nanomedicine* **2018**, 13, 4493. doi:10.2147/IJN.S167285

[36] Eissa, M. M., El-Moslemany, R. M., Ramadan, A. A., Amer, E. I., El-Azzouni, M. Z., El-Khordagui, L. K. *PloS one* **2015**, 10, e0141788. doi:10.1371/journal.pone.0141788

[37] El-Moslemany, R. M., Eissa, M. M., Ramadan, A. A., El-Khordagui, L. K., El-Azzouni, M. Z. *Acta tropica* **2016**, 159, 142-148. doi:10.1016/j.actatropica.2016.03.038

[38] Kolenyak-Santos, F., Garnero, C., De Oliveira, R. N., De Souza, A. L., Chorilli, M., Allegretti, S. M., Longhi, M. R., Chaud, M. V., Gremião, M. P. Journal of Nanoscience and Nanotechnology **2015**, 15, 761-772. doi:10.1166/jnn.2015.9186

[39] Zoghroban, H. S., El-Kowrany, S. I., Aboul Asaad, I. A., El Maghraby, G. M., El-Nouby, K. A., Abd Elazeem, M. A. *Parasitology research* **2019**, 118, 219–234. doi:10.1007/s00436-018-6132-z

[40] Amer, E. I., Abou-El-Naga, I. F., Boulos, L. M., Ramadan, H. S., Younis, S. S. *Biomedica: revista del Instituto Nacional de Salud* **2022b**, 42, 67–84. doi:10.7705/biomedica.5913

[41] Amer, E. I., El-Azzouni, M. Z., El-Bannan, R. T., Shalaby, T. I., El-Achy, S. N., Gomaa, M. M. *Acta Tropica* **2022a**, 226, 106231. doi:10.1016/j.actatropica.2021.106231

[42] Gonzalez, M. A., Ramírez Rigo, M. V., González Vidal, N. L. *AAPS PharmSciTech* **2019**, 20, 1-12. doi:10.1208/s12249-019-1548-z

[43] Cong, Z., Shi, Y., Peng, X., Wei, B., Wang, Y., Li, J., Li, J., Li, J. *Drug Development and Industrial Pharmacy* **2017**, 43, 558-573. doi:10.1080/03639045.2016.1270960

[44] Lima, L. I., Py-Daniel, K. R., Guimarães, M. A., Muehlmann, L. A., Mafud, A. C., Mascarenhas, Y. P., de Moraes, J., Leite, J. R. S. A., Jiang, C-S., Azevedo, R. B., Figueiro Longo, J. P. Nanomedicine **2018**, 13, 689-702. doi:10.2217/nnm-2017-0308

[45] Araújo, S. C., de Mattos, A. C. A., Teixeira, H. F., Coelho, P. M. Z., Nelson, D. L., de Oliveira, M. C. *International Journal of Pharmaceutics* **2007**, 337, 307-315. doi:10.1016/j.ijpharm.2007.01.009

[46] Aly, N., Husseein, A., Emam, H., Rashed, G. *Parasitologists United Journal* **2017**, 10, 44-51. doi:10.21608/PUJ.2017.4736

[47] Xavier, E. S., De Souza, R. L., Rodrigues, V. C., Melo, C. O., Roquini, D. B., Lemes, B. L., Wilairatana, P., Oliveira, E. E., de Moraes, J. *Frontiers in Pharmacology* **2022**, 13. doi:10.3389/fphar.2022.917363

[48] El-Feky, G. S., Mohamed, W. S., Nasr, H. E., El-Lakkany, N. M., Seif el-Din, S. H., Botros, S. S. Antimicrobial Agents and Chemotherapy **2015**, 59, 3501-3508. doi:10.1128/AAC.04875-14

[49] Bayaumy, F. E., Darwish, A. S. Materials Science and Engineering: C **2016**, 59, 717-730. doi:10.1016/j.msec.2015.10.074

[50] Darwish, A. S., Bayaumy, F. E., Ismail, H. M. Materials Science and Engineering: C **2018**, 93, 996-1011. doi:10.1016/j.msec.2018.09.007

[51] Yang, R., Zhang, T., Yu, J., Liu, Y., Wang, Y., He, Z. *Asian journal of pharmaceutical sciences* **2019**, 14, 321-328. doi:10.1016/j.ajps.2018.06.001

[52] Tawfeek, G. M., Baki, M. H. A., Ibrahim, A. N., Mostafa, M. A. H., Fathy, M. M., Diab, M. S. E. D. M. *Parasitology research.* **2019**; 118, 3519-3533. doi:10.1016/j.ajps.2018.06.001

[53] Mokbel, K. E. D. M., Baiuomy, I. R., Sabry, A. E. H. A., Mohammed, M. M., El-Dardiry, M. A. *Scientific reports* **2020**, 10, 1-9. doi:10.1038/s41598-020-72901-y

[54] Abou El-Nour, M. F., Kenawy, S. H., El-Bassyouni, G. T., Hamzawy, E. M. A. Advanced Pharmaceutical Bulletin **2021**, 11, 68. doi:10.34172/apb.2021.004

[55] Dkhil, M. A., Khalil, M. F., Diab, M. S., Bauomy, A. A., Santourlidis, S., Al-Shaebi, E. M., Al-Quraishy, S. *Saudi Journal of Biological Sciences* **2019**, 26, 1468-1472. doi:10.1016/j.sjbs.2018.02.008

[56] Dkhil, M. A., Bauomy, A. A., Diab, M. S., Wahab, R., Delic, D., Al-Quraishy, S. *Parasitology research* **2015b**, 114, 3711-3719. doi:10.1007/s00436-015-4600-2

[57] Dkhil, M. A., Khalil, M. F., Diab, M. S., Bauomy, A. A., Al-Quraishy, S. *Saudi Journal of Biological Sciences* **2017** 24, 1418-1423. doi:10.1016/j.sjbs.2016.12.017

[58] Dkhil, M. A., Bauomy, A. A., Diab, M. S., Al-Quraishy, S. *Biomed Res* **2016a**, 27, 214-219. doi:10.3967/bes2016.104

[59] Dkhil, M. A., Khalil, M. F., Bauomy, A. A., Diab, M. S., Al-Qura, S. *Biomedical and environmental sciences* **2016b**, 29, 773-781.

[60] Moustafa, M. A., Mossalem, H. S., Sarhan, R. M., Abdel-Rahman, A. A., Hassan, E. M. Parasitology research **2018**, 117, 3867-3880. doi:10.1007/s00436-018-6093-2

[61] Bauomy, A. A. *Environmental Science and Pollution Research* **2020**, 27, 18699-18707. doi:10.1007/s11356-020-08356-5

[62] El-Shorbagy, A., Gamil, I. S., Mohey, M. A., Nady, S. *Nanomedicine Journal* **2019**, 6. doi:10.22038/NMJ.2019.06.003

[63] Khalil, L. M., Azzam, A. M., Mohamed, H. A., Nigm, A. H., Taha, H. A., Soliman, M. I. Journal of the Egyptian Society of Parasitology **2018**, 48, 363-368. doi:10.12816/0050443

[64] Dkhil, M. A., Bauomy, A. A., Diab, M. S., Al-Quraishy, S. *International journal of nanomedicine* **2015a** 10, 7467. doi:10.2147/IJN.S97622

[65] Abd El Wahab, W. M., El-Badry, A. A., Mahmoud, S. S., El-Badry, Y. A., El-Badry, M. A., Hamdy, D. A. *PLoS Neglected Tropical Diseases* **2021**, 15, e0009423. doi:10.1371/journal.pntd.0009423
